# Benzodiazepine Sedation and Postoperative Neurological Deficits after Awake Craniotomy for Brain Tumor – An Exploratory Retrospective Cohort Study

**DOI:** 10.3389/fonc.2022.885164

**Published:** 2022-04-20

**Authors:** Eric Plitman, Tumul Chowdhury, Gabriel Paquin-Lanthier, Hirokazu Takami, Sudhakar Subramaniam, Kok Weng Leong, Abigail Daniels, Mark Bernstein, Lashmi Venkatraghavan

**Affiliations:** ^1^ Department of Anesthesia and Pain Management, Toronto Western Hospital, University Health Network, University of Toronto, Toronto, ON, Canada; ^2^ Division of Neurosurgery, Toronto Western Hospital, University Health Network, University of Toronto, Toronto, ON, Canada

**Keywords:** benzodiazepine, benzodiazepine sedation, midazolam, awake craniotomy, neurological symptoms, neurological deficits, brain tumor

## Abstract

An awake craniotomy is a common neurosurgical procedure for excising brain tumor(s) located near or in eloquent areas. The use of benzodiazepine (BZD) for sedation in some patients with neuropathological conditions (e.g., stroke, brain tumors) has been previously linked with re-appearance of neurological deficits including limb incoordination, ataxia, and motor weakness, resulting in complications for the patient along with procedural challenges. Whether or not these findings can be extrapolated to patients undergoing brain tumor resection is largely unknown. The current work primarily sought to compare neurological outcome(s) in the immediate postoperative period between BZD-free and BZD-based sedation techniques in patients undergoing awake craniotomy. Using a database composed of awake craniotomies conducted within a single center and by a single surgeon, patients were retrospectively classified based on midazolam administration into BZD-free sedation (n=125) and BZD-based sedation (n=416) groups. Patients from each group were matched based on age, sex, tumor location, tumor grade, preoperative neurological deficits, non-operative BZD use, and Karnofsky Performance Scale scores, resulting in 108 patients within each group. Postoperative neurological deficits were recorded. Logistic regression analyses were conducted comparing postoperative neurological deficits between the matched groups. Postoperative neurological deficits were more prevalent within the BZD-based sedation group compared to the BZD-free sedation group (adjusted odds ratio (aOR)=1.903, 95% CI=1.018-3.560, p=0.044). In addition, subgroup analysis of the matched cohort showed a relationship between preoperative neurological symptoms and postoperative neurological deficits in the BZD-based sedation group (aOR=3.756, 95% CI=1.390-10.147, p=0.009). Our findings support the notion that the increased incidence of postoperative neurological deficits with BZD sedation may in part be related to the unmasking of preoperative neurological deficits. Further studies are required to confirm this phenomenon.

## Introduction

Awake craniotomy is often indicated for resection of brain tumors that are located in/near eloquent areas of the brain to maximize the extent of surgical resection and to minimize the incidence of postoperative neurological deficits (1, 2). Recent meta-analyses have shown that awake craniotomy with intraoperative stimulation mapping is associated with decreased neurological morbidity in patients with high-grade glioma compared to resection under general anesthesia (3, 4). Previous work has shown that the incidence of new postoperative neurological deficits following awake craniotomy can be up to 13%, of which up to 4.5% can be permanent (5). Further, the presence of postoperative neurological deficits has been shown to be associated with preoperative deficits (6).

The success of an awake craniotomy is dependent on a variety of factors, including patient selection, preparation, and cooperation, as well as perioperative surgical and anesthesia care. Choice of anesthesia agents and their careful titration plays a significant role in the success of awake craniotomy. Among the anesthetic agents, benzodiazepines (BZD) are still being used for their amnestic and anxiolytic properties (1, 2, 7). Association between the choice of anesthetic agents and postoperative neurological deficits has not yet been explored extensively. A previous review by our group has shown that BZDs and opioid sedation can be associated with transient unmasking or worsening of neurological deficits in patients with previous brain injury (8). The incidence of re-emerging neurological deficits following the administration of anesthetic agents (i.e. BZDs, opioids, anesthetic induction agents, and other sedatives) was found to be as high as 46.3%; notably, this included an unmasking and/or a worsening of pre-existing neurological deficits (8). Further, in this review, 44% of patients experienced unmasking of neurological deficits after the administration of BZDs, opioids, or other sedatives (8). Interestingly, some of these deficits appear to be reversed by BZD and opioid antagonism using flumazenil and naloxone, respectively (8).

The current work sought to compare neurological outcomes in the immediate postoperative period between BZD-free and BZD-based sedation techniques in patients undergoing awake craniotomy. Further, to explore whether this phenomenon was related to a re-emergence of previous neurological deficits, we tested the relationship between preoperative and postoperative neurological deficits following the use of BZD-free and BZD-based sedation techniques in patients undergoing awake craniotomy. We hypothesized that BZD-based sedation would be associated with increased postoperative neurological deficits and re-emergence of previous neurological deficits would be greater following the use of BZD-based sedation techniques.

## Materials and Methods

### Study Design

We conducted a retrospective study using a database composed of all awake craniotomies for resection of brain lesions conducted within a single center and by a single surgeon over a 16-year period. This work received approval by the University Health Network institutional ethics board (#18-6131 and #21-5410), which included waiver of informed patient consent. The methodology has previously been described in two studies by our group: one examining the risk factors for intraoperative seizures during awake craniotomy and one exploring the risk factors for intraoperative complications (9, 10). Briefly, a retrospective chart review was carried out to collect surgical, anesthetic, demographic, pathologic, and radiologic data at our institution between July 13th 2006 and December 31st 2018 from all patients over 18 years of age who underwent an awake craniotomy by a single neurosurgeon (M.B.) for resection or biopsy of a space-occupying brain lesion. The database was maintained prospectively.

Data were extracted from this electronic record database for the present work to investigate the influence of intraoperative BZD sedation on neurological outcomes in the immediate postoperative period through a retrospective cohort study design. Patients were separated into BZD-free and BZD-based sedation groups (henceforth referred to as BZD (–) and BZD (+) groups, respectively), after which patients from each group were matched on key variables.

### Anesthetic Management

As described in Paquin-Lanthier et al. (9), standard anesthesia practice for awake craniotomy at our institution is monitored anesthesia care with conscious sedation. All patients scheduled for awake craniotomy are evaluated in the preoperative clinic by an anesthesiologist to assess fitness for awake craniotomy and obtain the informed consent. Antiepileptic drugs are continued until surgery if applicable; however, it is not routine practice to administer prophylactic preoperative or intraoperative antiepileptic drugs at our institution. The Canadian Anesthesiology Society standard monitoring is used for all cases, while invasive hemodynamic monitoring (arterial line) is rarely used. Supplemental oxygen is delivered by nasal prongs with capnography monitoring. Invasive airway management is used only if mandated to manage an intraoperative complication (about 0.16% of all awake craniotomy cases). A combination of intravenous sedatives (midazolam and/or propofol and/or dexmedetomidine) and opioids (fentanyl and/or remifentanil) is administered for maintaining conscious sedation at the discretion of the anesthesiologist. The main change in anesthesia management during the study period was the introduction of dexmedetomidine into our practice in April 2012; notably, there was an increased tendency to use dexmedetomidine in the years 2012 to 2018. Other aspects of anesthesia management remained unchanged throughout the duration of the study.

### Surgical Procedure

The surgical approach used by the neurosurgeon (M.B.) in this study has been previously described (9–13). An awake craniotomy with intraoperative stimulation mapping is performed for brain lesions in/adjacent to the eloquent cortex according to preoperative imaging. Awake craniotomy without intraoperative mapping is used with lesions remote from eloquent regions to avoid general anesthesia and accelerate patient recovery. After initiation of conscious sedation, local anesthesia is administered as a circumferential field block over the surgical incision and at the pin sites; the head is then fixed in a Sugita head clamp. Image-guided frameless navigation is used in all cases. Intraoperative stimulation mapping is performed using a bipolar stimulator to identify eloquent cortical and subcortical structures. The initial stimulation intensity is set at 8 mA for all patients and increased to 10 or 12 mA if no eloquent structures are identified and higher intensity stimulation is deemed safe by the neurosurgeon. Electrocorticography is not used during functional mapping during awake craniotomy at our institution. Motor, sensory, and/or language functions are evaluated intraoperatively with standardized tests. Eloquent cortical and subcortical areas identified by positive mapping are avoided during tumor resection. The endpoint of resection was subtotal to gross total resection with safe limits based on the intraoperative mapping results and image guidance. This surgeon has used a similar approach since 1991 for patient selection and surgical technique, and it was not modified during the study period.

### Data Sources, Measurements, and Definitions

Details regarding the data sources have been previously described elsewhere (9). Briefly, the data sources were the neurosurgeon’s prospective database and the institutional electronic patient record (Quadra Med Corporation, Reston, VA). Documents reviewed included preoperative anesthesia consultations, anesthesia records, post-anesthesia care unit nursing records, clinical notes, medication history, radiologic records, pathology reports, surgical dictation notes, and clinic visit notes. Collection of surgical and anesthetic data was performed by two independent groups (H.T. and M.B. for surgical data and G.P.-L., S.S., A.D, and K.W.L., for anesthetic data) and compiled in Excel (Microsoft, Redmond, WA). Any discrepancy between surgical and anesthetic databases was resolved by consulting the electronic record for validation of data collected (G.P.-L.) and discussion with the senior author (L.V.). Patients with missing data for any of the key variables referred to above were excluded. Midazolam dose was extracted only for descriptive purposes.

### Data Analysis

In the current work, midazolam administration was extracted from the database and used to classify patients into BZD-free sedation [BZD (–)] and BZD-based sedation [BZD (+)] groups. Further, variables to be used for matching of BZD (–) and BZD (+) groups were selected on the basis of previously published literature and authors’ clinical experience. These variables included age, sex, tumor location (i.e. frontal, parietal, temporal, or others), World Health Organization classification of central nervous system tumors [i.e. grade 1, grade 2, grade 3, grade 4, or metastatic (14)], preoperative neurological deficits (identified in preoperative medical, surgical, or anesthetic records), non-operative use of a BZD, and Karnofsky performance score. Our primary outcome was the incidence of postoperative neurological deficits defined as new or worsening neurological symptoms in the immediate postoperative period that persisted until at least discharge from hospital. For this study, neurological deficits were defined as presence of one or more signs/symptoms including weakness, sensory deficit, aphasia/dysphasia, visual field deficit, or cranial nerve deficit.

### Statistical Analysis

Statistical analyses were conducted using R version 3.5.0 and SPSS Statistics 24. First, in the full sample, demographic and clinical characteristics were compared between BZD (–) and BZD (+) groups using independent-sample t tests for continuous variables and *χ^2^* tests for categorical variables. A logistic regression was performed with the presence of postoperative neurological deficits as the outcome variable and midazolam usage, age, sex, tumor location (frontal or other), tumor grade (metastatic or non-metastatic), preoperative neurological symptoms, non-operative BZD use, and Karnofsky performance score as the predictor variables. A statistical threshold of P<.05 was employed.

Next, to address the imbalance between BZD (–) and BZD (+) groups that existed in the whole cohort, matching procedures were performed in these groups using the *MatchIt_4.2.0* package. Patients from each group were matched based on age, sex, tumor location, tumor grade, the presence of preoperative neurological deficits, non-operative BZD use, and Karnofsky Performance Scale scores. Categorical variables such as sex, tumor location, tumor grade, the presence of preoperative neurological deficits, and non-operative use of a BZD were matched with exact matching, whereas continuous variables such as age and Karnofsky performance score were matched with nearest neighbor matching. This rendered 108 patients within each of the BZD (–) and BZD (+) groups. Within the subset of patients who had a corresponding match, a logistic regression was performed with the presence of postoperative neurological symptoms as the outcome variable and midazolam usage as the predictor variable. A statistical threshold of P<.05 was employed.

The relationship between preoperative neurological and postoperative neurological deficits was tested using a logistic regression with the presence of postoperative neurological deficits as the outcome variable and the presence of preoperative neurological deficits as the predictor variable. This was performed separately in the whole cohort, in the matched cohort, and in each of the BZD (–) and BZD (+) groups. A statistical threshold of P<.05 was employed.

## Results

### Demographic and Clinical Characteristics

Five hundred and forty-one patients were included from the original database. Of these patients undergoing awake craniotomy, 125 had BZD-free sedation and 416 received BZD-based sedation. In the whole cohort, there were no differences in demographic and clinical variables, including age, sex, tumor location, tumor grade, preoperative neurological symptoms, non-operative BZD use, and Karnofsky performance score, between the groups (**Table 1**).

**Table 1:** Clinical and demographic characteristics of study participants.

	Whole Cohort		Matched Cohort	
	BZD (–)	BZD (+)	BZD (–)	BZD (+)
**n**	125	416	108	108
**Age [mean (SD)] (years)**	57.94 (15.78)	55.09 (15.64)	57.68 (15.69)	46.15 (14.46)
**Sex = Female n (%)**	52 (41.6)	195 (46.9)	46 (42.6)	46 (42.6)
**Tumor Location n (%)**				
*Frontal*	58 (46.4)	202 (48.6)	53 (49.1)	53 (49.1)
*Parietal*	23 (18.4)	90 (21.6)	21 (19.4)	21 (19.4)
*Temporal*	33 (26.4)	87 (20.9)	26 (24.1)	26 (24.1)
*Other*	11 (8.8)	37 (8.9)	8 (7.4)	8 (7.4)
**Tumor Grade n (%)**				
*Grade 1*	3 (2.4)	17 (4.1)	0 (0.0)	0 (0.0)
*Grade 2*	13 (10.4)	35 (8.4)	11 (10.2)	11 (10.2)
*Grade 3*	19 (15.2)	69 (16.6)	17 (15.7)	17 (15.7)
*Grade 4*	50 (40.0)	143 (34.4)	48 (44.4)	48 (44.4)
*Metastatic*	40 (32.0)	152 (36.5)	32 (29.6)	32 (29.6)
**Preoperative Neurological Deficits = Yes n (%)**	76 (60.8)	225 (54.1)	69 (63.9)	69 (63.9)
**Preoperative Benzodiazepine Use = Yes n (%)**	13 (10.4)	38 (9.1)	3 (2.8)	3 (2.8)
**Karnofsky Performance Score [mean (SD)]**	77.36 (13.08)	77.33 (13.83)	77.31 (12.80)	74.17 (12.69)
**Postoperative Neurological Deficits = Yes n (%)**	23 (18.4)	77 (18.5)	21 (19.4)	34 (31.5)
**Repeat Craniotomy = Yes n (%)**	27 (21.6)	75 (18.0)	26 (24.1)	29 (26.9)
**Midazolam Dose [mean (SD)] (mg)**	–	1.65 (0.85) *	–	1.72 (0.81) *

BZD, benzodiazepine; n, number; SD, standard deviation.

^*^One participant missing data.

### Relationship Between BZD Sedation and Postoperative Neurological Deficits

In the full sample, postoperative neurological deficits were identified in 100/541 subjects (18.5%), of which 77/416 were in the BZD (+) group (18.5%) and 23/125 were in the BZD (–) group (18.4%). Controlling for age, sex, tumor location, tumor grade, preoperative neurological symptoms, non-operative BZD use, and Karnofsky performance score, no relationship was identified between intraoperative BZD usage and postoperative neurological deficits (**Table 2**). However, relationships were identified between preoperative neurological deficits and postoperative neurological deficits as well as frontal location of tumor and postoperative neurological deficits.

**Table 2:** Logistic regression analyses of predictors for postoperative neurological symptoms.

	aOR	95% CI	*p*
**Midazolam Usage**	1.009	0.591-1.723	0.974
**Age**	0.987	0.972-1.001	0.076
**Female Sex**	1.316	0.833-2.077	0.239
**Preoperative Neurological Deficits**	2.194	1.233-3.902	0.008*
**Preoperative Use of Benzodiazepine**	1.205	0.586-2.475	0.612
**Frontal Location of Tumor**	2.363	1.473-3.792	<0.001*
**Metastatic Tumor**	0.606	0.367-1.000	0.050
**Karnofsky Performance Score**	0.992	0.971-1.013	0.433

aOR, adjusted odds ratio; CI, confidence interval.

*Denotes statistical significance.

**Figure 1:**
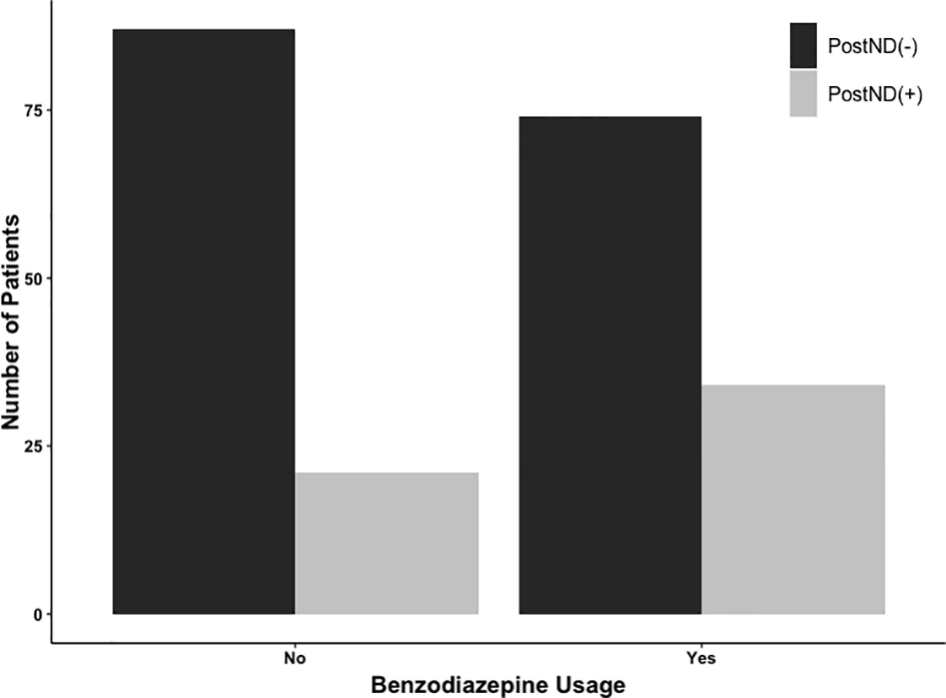
Number of patients undergoing awake craniotomy procedure that experienced postoperative neurological deficits (PostNDs) stratified by intraoperative usage of midazolam for sedation. In the benzodiazepine-based sedation [BZD (+)] group, 34 patients (34/108; 31.5%) experienced PostNDs, whereas 21 patients (21/108; 19.4%) experienced PostNDs in the benzodiazepine-free sedation [BZD (–)] group.

Subsequently, matching procedures were performed. Matching procedures rendered 108 matches between BZD (–) and BZD (+) groups (**Table 1**). In the matched cohort, 55/216 (25.5%) had postoperative neurological deficits; 34/108 (31.5%) were in the BZD (+) group and 21/108 (19.4%) were in the BZD (–) group (**Figure 1**). Logistic regression analyses within this subset of patients demonstrated an association between midazolam usage and postoperative neurological deficits (adjusted OR=1.90, 95% CI=1.02-3.56, p=0.04).

### Relationship Between Preoperative Neurological and Postoperative Neurological Deficits

**Figure 2:**
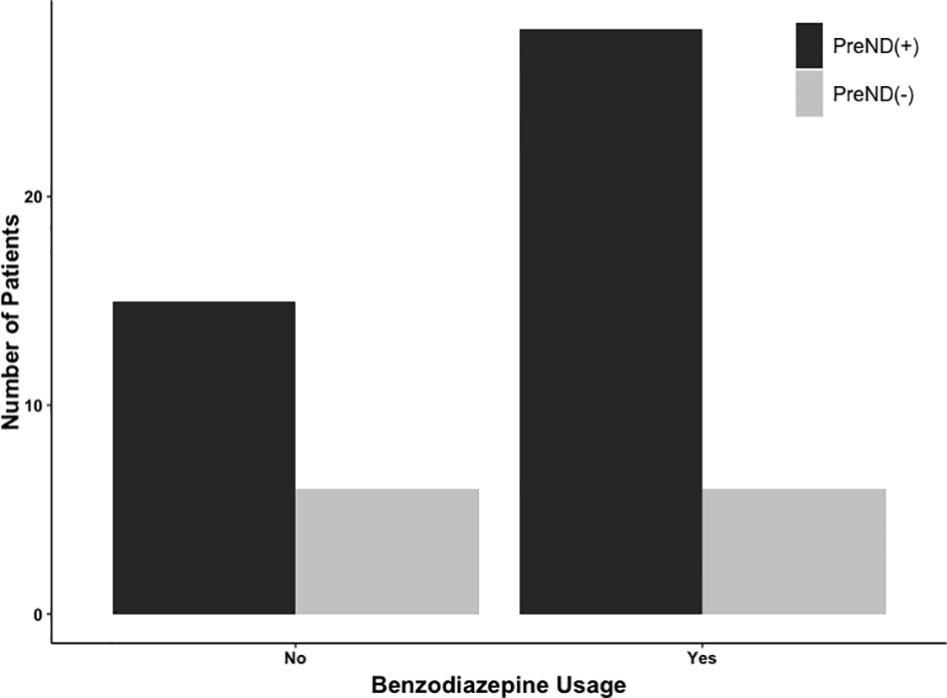
Number of patients undergoing awake craniotomy procedure that experienced postoperative neurological deficits stratified by preoperative neurological deficits (PreNDs). In the matched cohorts, 69 patients each had PreNDs in both benzodiazepine-based [BZD (+)] sedation and benzodiazepine-free [BZD (–)] sedation groups. In the BZD (–) group, 15 patients (15/69; 21.7%) patients developed postoperative neurological deficits and in the BZD (+) group, 28 patients (28/69; 40.6%) developed postoperative neurological deficits.

The number of patients with preoperative neurological and postoperative neurological deficits is presented in **Table 3**. In the whole cohort, 301/541 patients (55.6%) had preoperative neurological deficits; 225/416 were in the BZD (+) group (54.1%) and 76/125 were in the BZD (–) group (60.8%). In the matched cohort, 138/216 patients (63.9%) had preoperative neurological deficits, with 69/108 (63.9%) patients in each of the BZD (+) and BZD (–) groups (**Table 3**, **Figure 2**). Preoperative neurological deficits were related to postoperative neurological deficits in the whole cohort (adjusted OR=2.01, 95% CI=1.26-3.19, p=0.003) and the matched cohort (adjusted OR=2.49, 95% CI=1.22-5.08, p=0.01).

**Table 3:** Relationships between preoperative neurological symptoms and postoperative neurological symptoms.

	Whole Cohort (n=541)		Matched Cohort (n=216)		BZD (–) (n=108)		BZD (+) (n=108)	
	Preoperative Neurological Deficits							
	Yes	No	Yes	No	Yes	No	Yes	No
**n**	301	240	138	78	69	39	69	39
**Postoperative Neurological Deficits n (%)**								
Yes	69 (22.9)	31 (12.9)	43 (31.2)	12 (15.4)	15 (21.7)	6 (15.4)	28 (40.6)	6 (15.4)
No	232 (77.1)	209 (87.1)	95 (68.8)	66 (84.6)	54 (78.3)	33 (84.6)	41 (59.4)	33 (84.6)

BZD, benzodiazepine; n, number.

To further explain these findings, subgroup analyses of the matched cohort were performed. A relationship was identified between preoperative neurological symptoms and postoperative neurological symptoms in the BZD (+) group (adjusted OR=3.76, 95% CI=1.39-10.15, p=0.01), whereas no such relationship was found in the BZD (–) group (adjusted OR=1.53, 95% CI=0.54-4.33, p=0.42).

## Discussion

The present study sought to examine the impact of intraoperative BZD sedation on postoperative neurological deficits in patients undergoing awake craniotomy. Subsequent to matching on several key variables, a greater proportion of patients within the BZD (+) group was identified to have postoperative neurological deficits compared to patients within the BZD (–) group. Further, a relationship between preoperative and postoperative neurological deficits was identified in the BZD (+) group. This is one of the initial studies to match BZD (+) and BZD (–) groups on critical clinical and demographic variables that may impact postoperative neurological deficits (8).

The results from the current study suggest that BZD use for sedation in awake craniotomy procedures is associated with a greater likelihood of postoperative neurological deficits. This assertion is supported by findings from previous literature. One previous study reported that BZD use was a trigger for post-stroke recrudescence (15). Further, akin to the present work, transient worsening has been noted in patients with space-occupying lesions or an ischemic event specifically with midazolam usage (16–19). Moreover, there is evidence to support a phenomenon whereby neurological deficits associated with space-occupying lesions or ischemic events from which patients recovered are unmasked by BZD sedation. Previous studies have reported upon an association between midazolam use and transient unmasking of past neurological deficits (17–21). Notably, one study demonstrated reversibility of worsened deficits by treatment with flumazenil (16). This body of literature has been summarized in greater detail within a recent systematic review on this topic (8).

**Figure 3:**
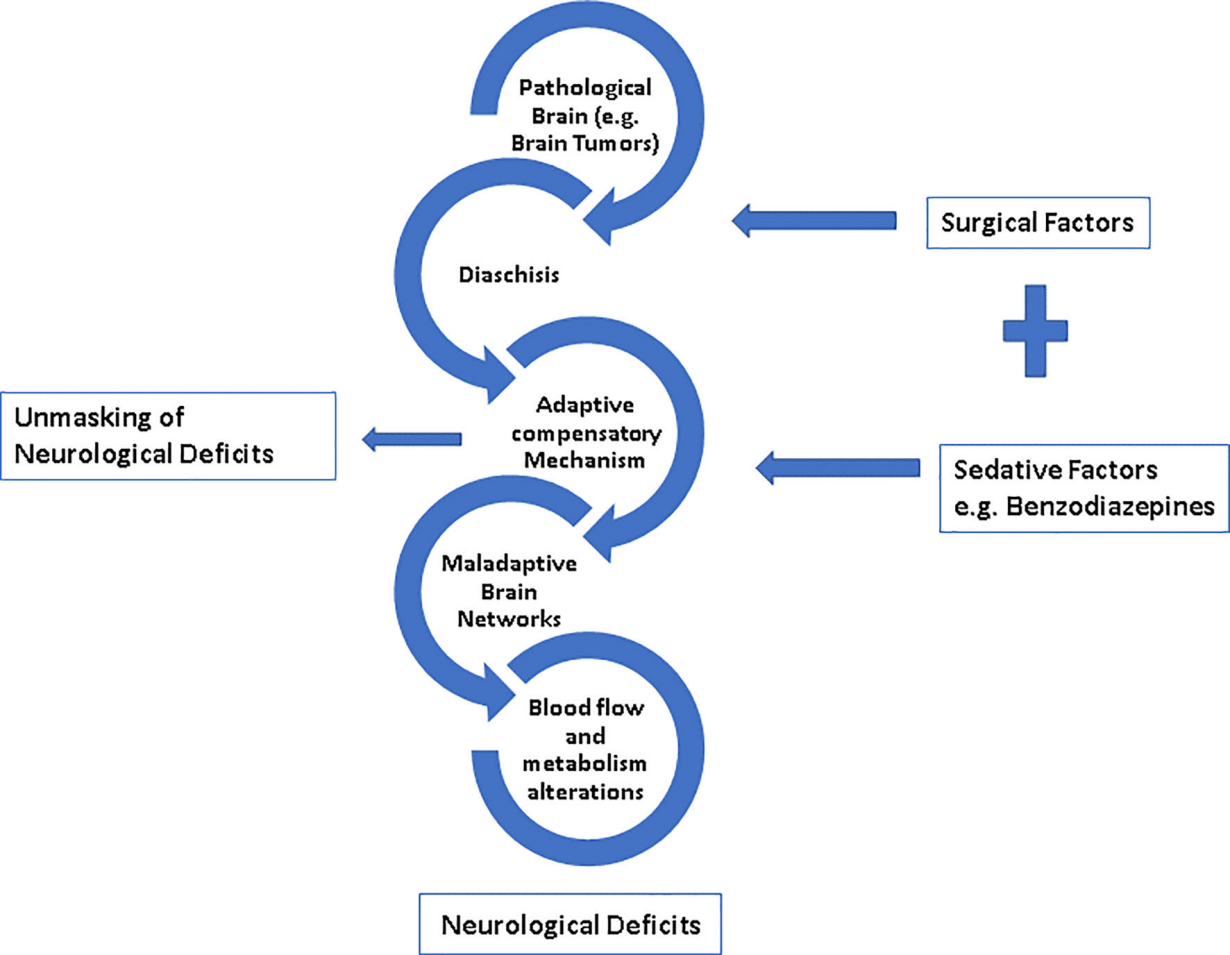
Plausible mechanisms for neurological deficits and unmasking of neurological deficits by sedatives.

The mechanism by which BZDs cause emergence or reemergence of neurological deficits is currently elusive. However, several hypotheses have been proposed in the literature (**Figure 3**) and greater detail is presented within Rizk et al. (8). A common theme amongst putative mechanisms is neural reorganization, encompassing structural and/or functional alterations following the original neurologic event. From a structural perspective, changes following cerebral insult due to the presence of a brain tumor have been purported to alter intracortical connectivity (8, 16, 19). Similarly, functional alterations to regional blood flow and metabolism may lead to increased delivery, slower washout, and/or increased metabolic suppression of BZDs (8, 19). Furthermore, previous cerebral insult may alter receptor density or functionality, increase the responsiveness of neurons to medications, and/or decrease redundancy in neuronal numbers (8, 17, 19). It deserves mention that, in the current work, groups were matched based on the presence of preoperative neurological deficits. However, irrespective of stratification by BZD sedation, the incidence of postoperative neurological deficits was greater in patients with preoperative neurological deficits, and this effect was further magnified in the BZD (+) group. Thus, our findings support the notion that the increased incidence of postoperative neurological deficits with BZD sedation may in part be related to the unmasking of preoperative neurological deficits.

The findings from the current work may have implications for clinical practice. BZDs appear to carry heightened risk for postoperative neurological deficits, which can lead to important complications as well as procedural challenges. As such, their usage for sedation should be carefully considered by multidisciplinary care teams, particularly in patients with a preoperative history of neurological deficits. Further, preoperative discussions with patients regarding the risks and benefits of these agents are warranted.

The present study has several limitations. First, the retrospective study design relies on data entry in the form of clinical detection and documentation by the neurosurgeon and anesthesiologist. As a result, postoperative neurological deficits may have been missed and data may be missing, incomplete, or inconsistent. Second, despite all cases within the database being led by one neurosurgeon, several anesthesiologists, fellows, and residents were involved in the care of these patients. However, most of the cases were performed by the same group of anesthesiologists and the protocol was very similar with minimal variations. Third, given that the current work was conducted at only a single center, its external validity may be limited. Fourth, the present study lacked long-term follow-up. As a result, we were unable to assess the timing of the identified neurological deficits, whether neurological deficits were transient or permanent, and any pharmacological and/or interventional reversibility of observed deficits. Fifth, despite having employed rigorous matching procedures, we cannot discount the influence of other confounders such as the sedation technique employed. Sixth, stringent and robust criteria for delineating neurological deficits could not be employed; however, the institutional standard practice of pre and postoperative neurological assessment might partially offset this limitation. Finally, the lack of volumetric data in the current work prevents an analysis exploring the relationship between extent of resection and BZD use; however, the use of a standard surgical technique with one surgeon may in part mitigate this limitation.

Our findings support the notion that the increased incidence of postoperative neurological deficits with BZD sedation may in part be related to the unmasking of preoperative neurological deficits. Multi-center prospective studies are required to confirm this phenomenon. In addition, more studies are needed to explore how different anesthetic agents and sedatives alter the brain connectomes in patients with or without brain tumors.

## Data Availability Statement

The raw data supporting the conclusions of this article will be made available by the authors, without undue reservation.

## Ethics Statement

The studies involving human participants were reviewed and approved by University Health Network institutional ethics board (#18-6131 and #21-5410). Written informed consent for participation was not required for this study in accordance with the national legislation and the institutional requirements.

## Author Contributions

EP, GP-L, LV, and TC performed the study design. MB, HT, GP-L, SS, KL, AD, LV contributed to data collection. EP, GP-L, LV, and TC contributed to data analysis. EP, LV, and TC composed the manuscript. LV and TC supervised the study. All authors contributed, critically reviewed, and approved the submitted manuscript.

## Funding

Departmental funding only.

## Conflict of Interest

The authors declare that the research was conducted in the absence of any commercial or financial relationships that could be construed as a potential conflict of interest.

## Publisher’s Note

All claims expressed in this article are solely those of the authors and do not necessarily represent those of their affiliated organizations, or those of the publisher, the editors and the reviewers. Any product that may be evaluated in this article, or claim that may be made by its manufacturer, is not guaranteed or endorsed by the publisher.
